# Effect of a 6-Week Cycle of Nordic Walking Training on Vitamin 25(OH)D_3,_ Calcium-Phosphate Metabolism and Muscle Damage in Multiple Myeloma Patients–Randomized Controlled Trial

**DOI:** 10.3390/jcm11216534

**Published:** 2022-11-03

**Authors:** Olga Czerwińska-Ledwig, David H. Vesole, Anna Piotrowska, Joanna Gradek, Wanda Pilch, Artur Jurczyszyn

**Affiliations:** 1 Department of Chemistry and Biochemistry, Institute of Basic Sciences, Faculty of Motor Rehabilitation, University of Physical Education in Kraków, 31-571 Krakow, Poland; 2 Myeloma Division, John Theurer Cancer, Center at Hackensack Meridian School of Medicine, Hackensack, NJ 07601, USA; 3 Department of Athletics, Institute of Sports Sciences, Faculty of Physical Education and Sport, University of Physical Education in Kraków, 31-571 Krakow, Poland; 4 Plasma Cell Dyscrasia Center, Department of Hematology, Faculty of Medicine, Jagiellonian University Medical College, 31-501 Krakow, Poland

**Keywords:** multiple myeloma, physical activity, vitamin D, muscle damage, calcium-phosphate metabolism

## Abstract

Introduction: Multiple myeloma (MM) is a hematological malignancy affecting older adults. One of the most common myeloma-defining events is the development of symptomatic lytic bone disease. The serum concentrations of calcium (Ca), inorganic phosphorus (P), and vitamin 25(OH)D_3_ in the serum reflect bone metabolism. An enzyme lactate dehydrogenase (LDH) is a marker of muscle damage, but its serum activity also has an important prognostic value in MM. Myoglobin (Mb) is a small protein present in muscles; its serum level increases when myocytes are damaged. Objectives: In this study, the impact of a 6-week Nordic walking (NW) exercise program on blood parameters related to calcium-phosphate metabolism and damage of skeletal muscles was assessed. Patients and methods: A total of 33 patients with MM in the remission stage, without cytostatic treatment, were allocated and randomly assigned to one of two groups: 17 in the training group (NW) and 16 in the control group (CG). All patients were supplemented per os with vitamin D3 and calcium carbonate daily and received zoledronic acid every 4 weeks (intravenous). Nordic walking training sessions took place 3 times a week for 6 weeks, 1 h each. Blood samples were drawn before and after the 6 weeks of training sessions to assess the serum concentrations of vitamin 25(OH)D_3_, P, Ca, Mb, and LDH. Results: Patients from the NW group showed a statistically significant decrease in mean serum myoglobin concentration (*p* = 0.018) and an increase in 25(OH)D_3_ (*p* < 0.001) and total Ca (*p* = 0.001) concentrations. There were no statistically significant changes in the results obtained in CG. Between groups, after 6 weeks, Mb serum concentration was significantly lower in NW (*p* = 0.041), and 25(OH)D_3_ was higher (*p* < 0.001) compared to CG. There was a correlation between the changes in myoglobin, phosphorus, 25(OH)D_3,_ and Ca concentrations after 6 weeks. Conclusions: NW training is a safe and beneficial form of physical exercise for patients with MM without inducing muscle damage. NW performed outside improves serum vitamin 25(OH)D_3_ concentration.

## 1. Introduction

Multiple myeloma (MM) is a plasma cell malignancy producing a monoclonal paraprotein, either an intact immunoglobulin or its fragment, which is detected by electrophoresis in the serum and/or urine [1]. MM accounts for 10–15% of all hematological malignancies and about 1–2% of all cancer cases [2]. It occurs predominantly in elderly people, the median age at diagnosis is 70 years, and 90% of patients are over 50 years old [1,3]. Over the past 18 years, 15 new drugs have been approved for the treatment of MM, resulting in almost doubling the median survival to over 55% at 5 years [4]. The goal of anti-myeloma therapy is to achieve a complete remission to the level of minimal residual disease. The frequency with which therapeutic strategies are utilized is dependent on their availability in individual countries [5].

Bone involvement in MM occurs in 80% of patients and is manifested as myeloma bone disease (MBD) [6]. It includes skeletal complications such as osteolytic lesions, bone pain, pathological fractures, and osteoporosis [1]. The formation of osteolytic lesions is associated with the disturbance of the balance between osteoblasts and osteoclasts; it is shifted towards the osteoclastic destruction of bone tissue resulting from the interaction of neoplastic plasma cells and their cytokines within the bone marrow microenvironment [7]. Osteolytic lesions rarely heal, but the process of their formation can be slowed or stopped by administering bisphosphonates or rank-ligand inhibitors [6]. In addition, bisphosphonates/rank-ligand inhibitors are effective in the treatment and prevention of hypercalcemia [1]. The successful treatment of MM by anti-myeloma agents also prevents the development of new MBD.

Patients with MM are candidates, even with MBD, for physical activities of low and moderate intensity [8]. In Poland, patients with MM do not have ready access to rehabilitation centers or private physical therapists [9]. A number of research studies have shown the beneficial effect of regular physical activity in patients with MM, with improvement in functional and psychological aspects [10,11,12], even if applied during MM therapy [13,14]. However, there are no reports assessing the impact of physical training on biochemical parameters in MM patients. Myoglobin (Mb) and lactate dehydrogenase (LDH) are myocyte damage markers often used in sports. LDH is an enzyme present in all cells, but its highest activity is observed in the cells characterized by a high level of energy metabolism: in skeletal and heart muscles, liver cells, neurons, and erythrocytes. Additionally, LDH serum activity is of prognostic importance in myeloma [1]. Myoglobin is a protein present in heart and skeletal muscle cells with corresponding increases in Mb level with muscle damage. Mb functions in the storage of oxygen in the muscle; it releases oxygen molecules when the partial pressure in the cell decreases. In addition, serum Mb concentration may be associated with deteriorating renal function with age [15]. The concentration of Mb in skeletal muscle cells increases as a result of regular exercise in an adaptive mechanism, which leads to an increase in the efficiency of oxygen delivery during exercise. Serum Mb levels rise in case of muscle damage, which leads to leakage of this protein from the cells. A high concentration of Mb may have a damaging effect on the glomeruli [16]; therefore, its control is indicated in patients with MM.

Physical activity affects bone metabolism in the mechanism of mechanotransduction which could be reflected in serum concentrations of bone turnover markers such as Ca [17]. The calcium-phosphate metabolism is regulated by vitamin D3, calcitonin, and parathyroid hormone. The decrease in blood Ca concentration stimulates the release of Ca from the mineralized osteoid within minutes by the action of parathyroid hormone and vitamin 1,25(OH)2D (calcitriol), which activates the release of Ca and P from the bone. In the kidney, there is an increase in the production of 1,25(OH)2D, leading to increased reabsorption of Ca ions and inorganic P in the renal tubules. Additionally, the intestinal absorption of Ca, P, and magnesium increases.

One of the most frequently recommended forms of physical activity for elderly patients and people with low tolerance to physical effort is marching with poles in the form of Nordic walking training [18]. Walking interventions are also recommended for people suffering from hematological malignancies [19], as well as patients after other types of cancer [20,21,22].

In light of the above facts, the aim of this study was to assess the effect of a 6-week Nordic walking training cycle on the blood concentrations of the active metabolite of calcidiol (25(OH)D_3_), total calcium (Ca), inorganic phosphorus (P), myoglobin (Mb) and lactate dehydrogenase (LDH) activity in MM patients in remission. We assumed that the training sessions would not negatively affect the parameters of calcium-phosphate metabolism and muscle damage parameters. Our hypothesis also assumed that 6 weeks of physical activity performed by patients in the open air could improve the serum concentration of the vitamin D metabolite.

## 2. Materials and Methods

### 2.1. Study Group

Fifty-seven MM patients in the Department of Hematology of Jagiellonian University Medical College in Krakow were recruited to participate in this study by their attending physicians. Thirty-three of them met the project’s inclusion criteria, did not meet the exclusion criteria, and agreed to participate in the study. The inclusion criteria were MM in remission, Eastern Cooperative Oncology Group (ECOG) scale: 0.1.2 (0—fully active, 1—restricted in physically strenuous activity but ambulatory and able to carry out work of a light or sedentary nature, 2—ambulatory and capable of all self-care but unable to carry out any work activities; up and about more than 50% of waking hours [23]), no contraindications to participate in health training, and no chronic respiratory diseases. The exclusion criteria were hypercalcemia, active infection requiring treatment, and any pre-existing injury of the limbs or trunk which would limit participation in the study.

**Figure 1:**
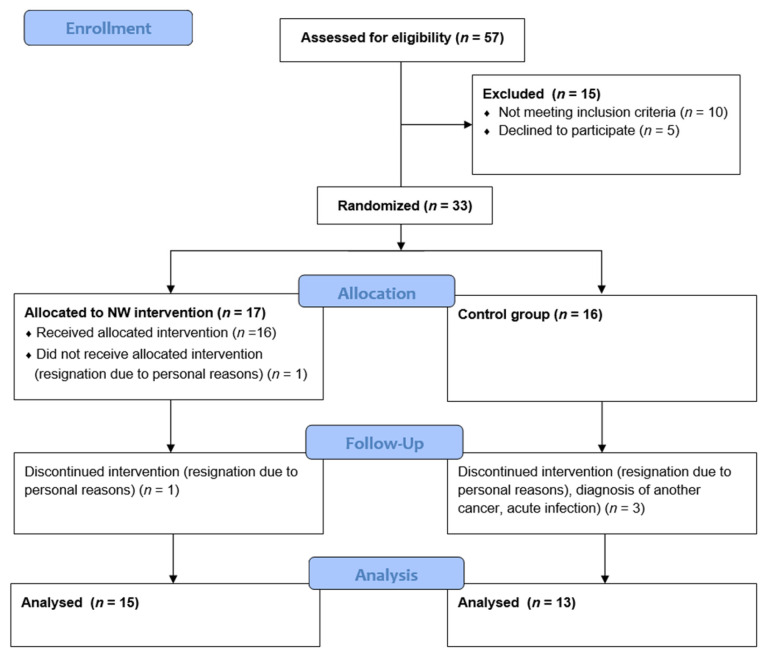
CONSORT 2010 patient flow diagram.

Subsequently, a permuted block randomization procedure (ratio 1:1) was performed using opaque envelopes. The envelopes were prepared by researcher 1, then, after closing, passed to researcher 2, who assigned participants to groups by drawing. Participants were randomly assigned to one of two groups: Nordic walking (NW; *n* = 17) and the control group (CG; *n* = 16). The NW group underwent a 6-week health walking training cycle; the CG did not have any formal planned physical activity. All patients were supplemented with 2000 U of vitamin D3 and 1000–1500 mg calcium carbonate daily and received 4 mg zoledronic acid i.v. every 4 weeks according to the current recommendations of the Polish Myeloma Working Group [1] and International Myeloma Working Group [6]. Fifteen patients completed the program in the NW group and thirteen in the CG group. The patient flow diagram is shown in Figure 1.

The patients were informed that participation in this study was voluntary, and they were acquainted with the details of the project. The study protocol was constructed according to the Declaration of Helsinki, and all participants gave their written consent. The procedures of this study were approved by the Bioethical Committee at the District Medical Chamber in Krakow (166/KBL/OIL/2018). This study is also registered as a clinical trial in ANZCTR (no: ACTRN12622000268741).

### 2.2. Study Protocol

Blood samples were obtained pre-study (in the NW group, 2 days before the beginning of training sessions) and at the completion of the study at 6 weeks. The determination of the concentrations of biochemical indices in the serum was performed in the medical diagnostic laboratory with the use of a Cobas 6000/8000 analyzer (Roche Diagnostics, Indianapolis, IN, USA). The concentrations of P and total Ca were determined using the colorimetric method. LDH activity was determined according to the UV test methodology. Vitamin 25(OH)D_3_ concentration was determined with the use of the electrochemiluminescence (ECLIA) method and myoglobin concentration with the use of the chemiluminescence method.

### 2.3. Nordic Walking Training

Nordic walking training sessions were performed 3 mornings a week during spring–summer at the Academy of Physical Education in Krakow by a qualified instructor for 6 weeks. The Nordic walking pole lengths were adjusted for each participant individually. The subjects were instructed in the correct Nordic walking technique, which was individually monitored and adjusted for each participant during the training. The maximum heart rate (HRmax) was calculated with the use of the Nes formula validated in elderly subjects [24]. The exercise intensity was fixed at a mean level (60–70% HRmax) and was constantly monitored during training sessions with the use of a sports tester (Polar, Kempele, Finland).

Each session consisted of a 10-min warm-up, including limb, general development, and dynamic stretching exercises. The planned duration of the Nordic walking was 45 min. The walking time, and thus, the distance, was extended with each training session until it reached a maximum of 45 min. At the end, there was a 5-min cooling period, during which the patients performed breathing and stretching exercises.

### 2.4. Statistical Analysis

Descriptive statistics (mean, standard deviation) were calculated for all variables, and the normality of the distribution was assessed (Shapiro–Wilk test) [24]. Student’s *t*-test for dependent groups was used to compare the results of the blood biochemical analyses in each group. The *t*-test for independent groups was used to compare the obtained results between the groups. For variables with other than normal type of a distribution, the Wilcoxon test and the Mann–Whitney U test were used. Pearson’s r correlations were also calculated for all tested parameters. The results *p* < 0.05 were considered statistically significant. Statistical analyses were performed using JASP 0.16.1 software (University of Amsterdam, Amsterdam, The Netherlands).

## 3. Results

### 3.1. Study Group Characteristics

The 6-week NW cycle was completed by 15 patients (8 women and 7 men); 13 patients (5 women and 8 men) completed the study as the control group (CG). A total of 5 patients (2 from NW and 3 from CG) did not complete the study due to personal reasons (3) or meeting the exclusion criteria during their participation in this research project (2). The attendance rate in the NW group was over 90%. Study group characteristics are shown in Table 1.

### 3.2. Serum Concentrations of Studied Parameters

The results of measurements of serum concentrations of biochemical parameters in study participants at baseline and after 6 weeks are shown in Table 2.

Serum Mb concentration after 6 weeks in the NW group decreased significantly from the initial level by an average of 6.1 μg/L (t = 2.687, *p* = 0.018). No statistically significant changes were observed in the CG group for this parameter. Between the groups, statistically significant changes were observed in the Mb concentration (t = 2.150, *p* = 0.041). No significant differences in groups and between groups were noted for LDH activity at both time points.

The concentration of 25(OH)D_3_ (calcidiol) in the NW group at baseline was within the normal range in 6 patients (between 30–80 ng/mL, mean: 37.75 ± 6.3 ng/mL), low (between 20–30 ng/mL, mean: 25.4 ± 2.5 ng/mL) in 4 patients, and the severely low in 5 patients (<20 ng/mL, mean: 13.2 ± 1.8 ng/mL). In the NW group, the concentration of calcidiol increased significantly in all subjects (t = −5.809, *p* < 0.001) on average by 9.9 ng/mL (to the mean concentration: 26.7 ± 3.8 ng/mL). After 6 weeks, in 10 subjects, the calcidiol levels either remained in the normal range (*n* = 6) or improved from low or severely low (4/9) (mean: 41.3 ± 9.8 ng/mL). Even after 6 weeks of NW training, 5 patients remained in the low range but none in the severely low range (mean for these patients: 26.7 ± 3.8 ng/mL). There were statistically significant changes between the groups in vitamin 25(OH)D_3_ concentration (t = −4.389, *p* < 0.001).

Concomitantly to calcidiol level changes, an increase in serum total Ca concentration was observed by an average of 0.08 mmol/L (t = −3.990, *p* = 0.001). There was a trend toward improvement in the total Ca concentration between groups (t = −1.917, *p* = 0.066). In contrast, there was not a significant change in P concentrations in either group. The concentrations of P and Ca in all subjects from both groups were within the reference values. In the control group, after 6 weeks, no statistically significant changes in 25-(OH)D_3_, Ca, P, Mb serum concentrations and LDH serum activity were found.

### 3.3. Correlations

**Figure 2:**
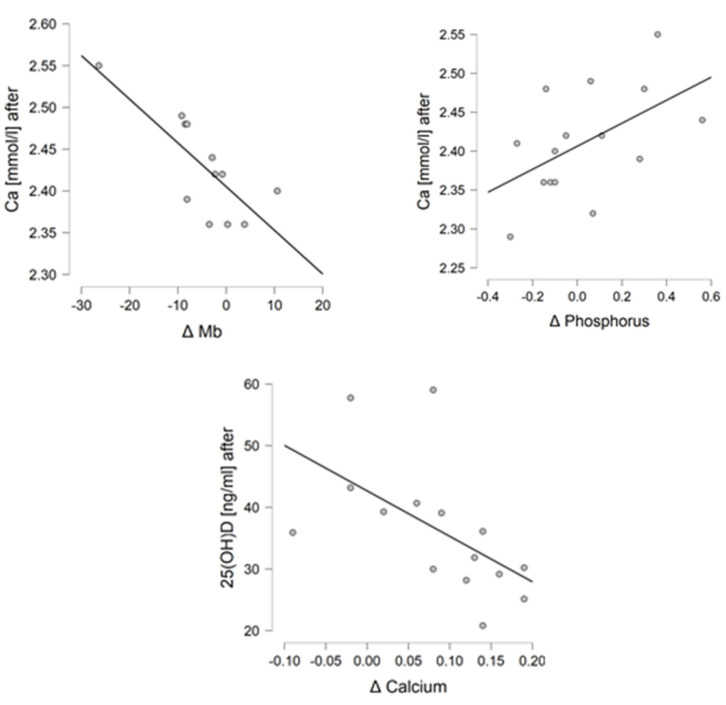
Correlations of statistically significant biochemical indices in the NW group.

**Figure 3:**
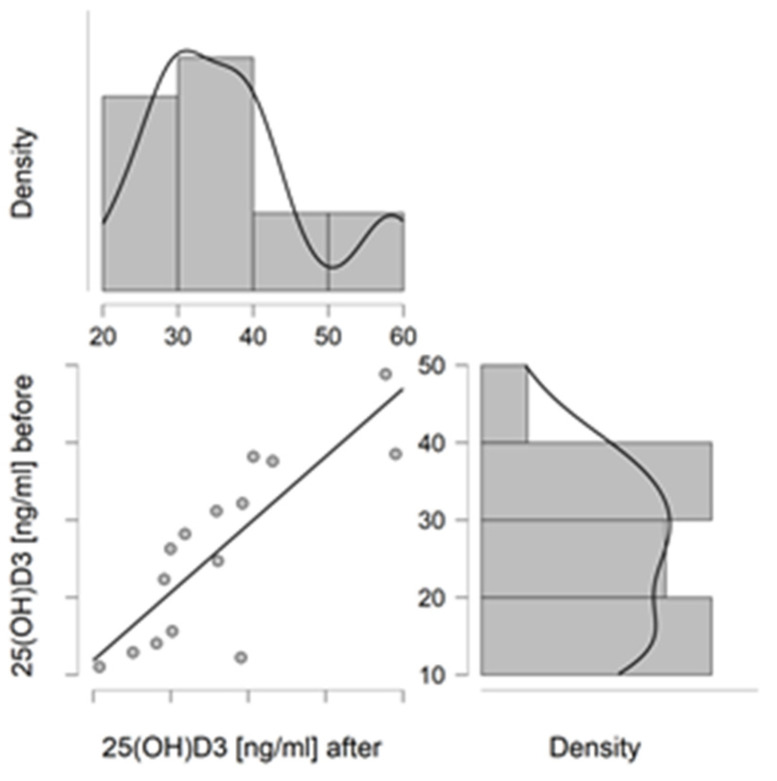
Correlation plot for 25(OH)D_3_ concentrations in patients from the NW group at baseline (before) and after 6 weeks of training (after).

In the NW group, a correlation was found for changes in the concentration of Mb and P with the concentration of Ca after 6 weeks (r = −0.773, *p* = 0.003, and r = 0.527, *p* = 0.043, respectively). Additionally, the concentration of Ca with the concentration of 25-(OH)D_3_ after 6 weeks of training (r = −0.560, *p* = 0.030) was correlated. For the remaining parameters, no statistically significant corrections were found. In the control group, no statistically significant correlation was found between the studied indices. There was no correlation between age and any of the parameters. Graphs showing statistically significant correlations are shown in Figure 2 and Figure 3.

## 4. Discussion

### 4.1. Biochemical Parameters of Skeletal Muscle Damage

#### 4.1.1. Lactate Dehydrogenase (LDH)

Changes in serum LDH activity depend on the type of training and the level of adaptation to training; it increases significantly when muscle damage occurs. Callegari et al. [25] compared changes in LDH activity after aerobic training with the intensity of 60%VO2max and 80%VO2max and aerobic training combined with resistance exercises. LDH activity measurements before and after training showed a statistically significant increase in all groups. Participation in NW may also affect LDH levels. Hagner-Derengowska et al. [26] reported on 32 post-menopausal women and showed that LDH activity in its participants decreased significantly after a cycle of 10-week NW training (184.3 vs. 175.7 IU/L). In our study, LDH activity increased slightly in the NW group, while in the control group, there was a minimal decrease in the activity of this enzyme. These changes, however, were not statistically significant, which allows us to conclude that the NW training stimulus was not strenuous enough to affect LDH activity. The lack of a statistically significant increase after the applied training cycle shows that the proposed training form is properly selected in terms of volume and intensity.

Elevated LDH activity in MM patients, after excluding other causes, may be associated with extensive disease or with the presence of extramedullary plasmacytoma(s) [1]. Teke et al. [27] presented a case of an MM patient in which LDH activity was within the reference range at the diagnosis but increased 27 times during progression and occurrence of extramedullary plasmocytomas. LDH activity level is a useful prognostic parameter in MM patients and is included in the revised international MM staging system (R-ISS) [1]. Gu et al., reported that both progression-free survival (PFS) (12.0 months versus 24 months, *p* < 0.001) and overall survival (OS) (15.5 months versus. 52.5 months, *p* = 0.030) were shorter compared to those with normal LDH levels [28]. In patients participating in our study, LDH activity levels were within the reference range throughout the entire duration of the project, which shows that the applied moderate-intensity training cycle is safe for MM patients in the remission stage.

#### 4.1.2. Myoglobin (Mb)

Initial Mb concentration in MM patients participating in our study was similar to the concentrations reported previously by Anesi et al., for healthy elderly subjects [15]. The mean Mb concentration in this population was 53.7 µg/L in men and 44.9 µg/L in women. In subjects taking part in our study, we observed a statistically significant decrease in the mean serum Mb concentration in the NW group as a result of participation in the NW (from 50.5 to 44.4 µg/L), while in the CG, a slight increase was observed (from 52.8 to 53.6 µg/L). Statistically significant differences were shown between these two groups after 6 weeks; serum concentration in subjects from the NW group was significantly lower than in the CG. Physical forces generated by gravity, locomotion, and exercise act on bone and muscle cells, which react by releasing a series of myokines and kinins [29]. Long-term changes caused by, e.g., repeated physical exercises induce adaptation: the tissue is restored and reorganized. This fact could explain a decrease in Mb concentration in our study group. Vitamin D supplementation may also affect post-exercise changes in the markers of muscle damage, such as myoglobin. Żebrowska et al. [30] showed in their work that post-exercise myoglobin serum concentration was lower in participants supplemented with 2000 UI of vitamin D 3 daily. In our study, higher vitamin D levels, as well as adaptation to exercise, may have caused a decrease in serum myoglobin levels from the NW group.

Physical exercise affects serum Mb concentration, which increases after a single exercise in proportion to its intensity, which was demonstrated by Cornish et al. [31] in 11 men over 65 years old. Their report shows a significant increase in serum Mb concentration after 3 h. In the case of low exercise intensity, it returned to the baseline values after 24 h, and in the case of moderate and high intensity, after 48 h. The blood collection after 6 weeks of the training cycle in our study was performed more than 48 h after the completion of the exercise cycle; hence, it had no effect on Mb concentration.

### 4.2. Bone Metabolism Indicators: Calcium-Phosphate Metabolism, Vitamin 25-(OH)D3

#### 4.2.1. Calcium (Ca) and Phosphorus (P)

Exercise affects the body’s ionic balance, including blood levels of Ca and P. Acute physical exercise causes a decrease in Ca concentration and an increase in serum inorganic P concentration. In a study by Karakukcu et al. [32], 32 healthy adolescent men rigorously undergoing boxing training showed a statistically significant increase in serum Ca concentration at 4 weeks from 9.62 mg/dL to 9.90 mg/dL (*p* < 0.001) and a slight increase in inorganic P from 4.56 mg/dL to 4.70 mg/dL was observed. In contrast, Kałużny et al. [33], in which 32 obese post-menopausal women underwent a 10-week cycle of NW training (30 sessions, 60 min each), a statistically significant decrease in serum total Ca concentration and a non-significant decrease in inorganic P concentration were shown.

In our study, similar relationships were observed; a statistically significant increase in serum total Ca concentration and a non-significant increase in inorganic P concentration after 6 weeks of NW training. However, the initial mean serum concentration values (Ca—2.33 mmol/L, P—0.99 mmol/L) of both groups in our study were lower than in one by Karakukcu et al. [32], which may be related to the age of the participants in our study. In the elderly, Ca absorption from the gastrointestinal tract is often deteriorated, and reabsorption in the renal tubules is impaired. In the study by Markiewicz-Żukowska [34] conducted on the population of 99 Polish seniors (mean age: 76 years), the mean serum total Ca concentration was (2.07 mmol/L); there were no gender differences for this parameter. Whereas our results are consistent with that reported by Karakukcu et al. [32], we do not know why Kaluzny et al. [33] reported the opposite results.

MM affects the calcium-phosphate balance; hypercalcemia may occur in approximately 15–20% of patients at diagnosis and is one of the CRAB diagnostic criteria [1]. Patients with MM may also develop hyperphosphatemia associated with renal failure and pseudohyphosphatemia associated with the interference of monoclonal protein in the IgG class with the method of determination of serum inorganic P [35,36]. Renal-related hypophosphatemia in patients with MM may be associated with acquired Fanconi syndrome, which is a rare complication of MM. It is caused by a reabsorption dysfunction in the proximal tubules [37]. It was shown in a study by Umeda et al. [38] that high levels of blood P are a negative prognostic factor, while for Ca levels, no significance was shown.

Renal impairment during MM is related to Ca-P balance disruption occurring in MBD. Kidneys regulate Ca serum level via the production of vitamin 1,25(OH)D_3_ (calcidiol). Bisphosphonate administration during MBD treatment helps to restore the balance between osteogenesis and bone tissue resorption [1]. Bisphosphonate–zolendronic acid was administered to all of the patients taking part in this project as a part of MDB control. The data obtained in our study is consistent with the results of Swenson et al. [39], which showed that physical activity in the form of a home-based exercise program did not prevent bone loss in breast cancer patients as effectively as zolendronic acid administration. Another study showed that physical activity in the form of treadmill exercises performed in rats in combination with zolendronic acid did not have a synergistic or additive effect [40]. All participants were receiving this drug before enrollment in the study, and it should be assumed that during the training period, its effect did not affect the concentration of Ca and P serum levels. The changes observed after the training cycle did not affect the effectiveness of this treatment; the observed changes in P and Ca concentrations were within the reference ranges. This indicates the safety of the applied physical activity for patients undergoing this type of therapy.

#### 4.2.2. Vitamin 25(OH)D_3_

Vitamin D is a fat-soluble vitamin. The serum concentration of vitamin D serum concentration is dependent on oral intake and skin synthesis [41]. Its reduced concentration is associated with the development of noncommunicable diseases (NCD) and various types of cancers [41]. It was also shown that the optimal serum level of vitamin D might contribute to lower post-exercise concentrations of biochemical indicators of muscle damage compared to subjects with its suboptimal concentration [42].

Serum 25(OH)D_3_ concentrations are seasonally dependent; they are higher in the spring/summer due to sunlight ultraviolet light exposure [41]. Pilch et al. [43] indicated a statistically significant increase in calcidiol concentration in women in NW training for a period of 12 weeks from March to May. The mean change in 25(OH)D_3_ concentration was +3.5 ng/mL and was lower than in this study (+9.9 ng/mL). Pilch et al. [44] also reported a decrease in 25(OH)D_3_ concentration in a 6-week cycle of NW training taking place in late autumn. The difference in the results is probably a reflection of the timing of the studies: May–July in our study versus autumn in the Pilch et al. study. The total solar irradiation time during our research period was longer than in the other studies. A significant increase in the concentration of 25(OH)D_3_, which was statistically significant, took place in our study and was most likely associated with an increase in exposure to UV radiation [41]. Increased exposure to UV radiation intensifies skin synthesis, which is the main source of vitamin D, apart from that taken with food [45]. According to the data supplied by the Institute of Meteorology and Water Management (Warsaw, Poland) in the study period, the sum of insolation for the city of Kraków and suburban areas in May was in the range of 140–160 h, in June 310–330 h, and 220–240 in July. The difference in insolation duration between the beginning and the end of the study period explains the increase in vitamin 25(OH)D_3_ concentration in the CG in our study. It is worth noting, however, that in the NW group, the increase in serum concentration of this vitamin was much higher.

Vitamin 25(OH)D_3_ concentration is also related to BMI. In obese individuals, the bioavailability of vitamin D is reduced due to its accumulation in adipose tissue. Wortsman et al. reported lower 25(OH)D_3_ levels in individuals with a BMI above 30 kg/m^2^ compared to the control group with a BMI below 25 kg/m^2^ [46]. In obese people, the bioavailability of vitamin D is reduced due to its accumulation in adipose tissue, which was demonstrated in people with a BMI above 30 kg/m^2^ compared to the control group with a BMI below 25 kg/m^2^ [46]. In our study, there was no correlation found between baseline vitamin 25(OH)D_3_ serum level and BMI.

### 4.3. Study Limitations and Strengths

This is the first study assessing the effect of a Nordic walking training cycle on the blood parameters of patients with MM in remission. This allows for unique results with practical implications. This type of physical activity can be safely recommended to patients in this stage of the disease. We selected only patients in the remission stage because of the lack of previous reports on the effect of physical activity on MM patients’ blood parameters.

Currently used therapies allow for achieving remission in a large number of patients, and the survival rate is constantly extending. Hence, research on health training allows us to learn more about the scope of safe physical activity in these patients who want to improve their physical capacity. As the moderate-intensity training applied in this study was proven to be safe for patients in MM remission, we suggest that in future studies, patients with active treatment could be included. However, in this case, the side effects of the therapy, such as fatigue and weakness, should also be taken into account. It is worth noting that in patients during anti-myeloma treatment, it is difficult to determine the maximum heart rate with the use of physiological tests, and calculating it from the formula does not reflect the actual capacity of the patient.

The small number of participants involved in this study was caused by limited access to MM patients as well as by the patients’ concerns about participating in a physical activity program. Due to the limited budget, this study was not supplemented with any imaging methods which should be included in future studies on this subject. Although we did not observe any end-related side effects during the duration of the program and in follow-up 3 weeks after its completion, a longer follow-up period on adverse bone events and with the use of medical imaging methods would also provide added value.

## 5. Conclusions

In summary, individually adapted moderate-intensity Nordic walking for 6 weeks, adjusted to the patient’s capacity, did not cause muscle damage assessed by serum Mb or LDH determinations but had a positive effect on the vitamin D serum concentration. Therefore, we conclude that NW training can be considered a safe and beneficial form of physical activity for patients with MM in terms of blood parameters related to MBD control. This has clinical significance and may indicate the usefulness of this form of exercise in improving the daily functioning of patients in multiple myeloma remission.

## Figures and Tables

**Table 1:** Study group characteristics.

	NW (*n* = 15)	CG (*n* = 13)
Age [years]	62.3 ± 8.5	63.7 ± 3.7
MM duration [months]	35.9 ± 14.8	35.6 ± 16.8
ASCT [*n* (%)]	15 (100%)	13 (100%)
Polyneuopathy [*n* (%)]	9 (60%)	6 (46%)
Bone pain [*n* (%)]	14 (93%)	12 (92%)
MBD [*n* (%)]	15 (100%)	12 (93%)
Anthropometrics:		
Body mass [kg]	164.0 ± 10.6	166.9 ± 6.3
Height [cm]	78.7 ± 10.8	79.4 ± 16.2
BMI [kg/m^2^]	29.3 ± 3.5	28.3 ± 4.5

Results shown as arithmetic mean ± standard deviation (SD). Statistically significant differences between groups were not observed. ASCT––autologous stem cell transplantation, BMI––body mass index, MBD––myeloma bone disease.

**Table 2:** Changes in average concentrations of biochemical indices in multiple myeloma patients after a cycle of 6-weeks of Nordic walking training.

	NW (*n* = 15)		CG (*n* = 13)	
	Baseline	After 6 Weeks	Baseline	After 6 Weeks
Mb [µg/L]	50.5 ± 22.6 *	44.4 ± 16.5 *^,^#	52.8 ± 21.3	53.6 ± 20.7 #
LDH [U/L]	200.8 ± 34.0	204.3 ± 36.8	200.3 ± 39.9	198.3 ± 35.8
Ca [mmol/L]	2.33 ± 0.07 *	2.40 ± 0.07 *	2.37 ± 0.14	2.39 ± 0.10
P [mmol/L]	0.99 ± 0.22	1.02 ± 0.17	0.97 ± 0.21	0.98 ± 0.20
25(OH)D_3_ [ng/mL]	26.3 ± 11.6 *	36.4 ± 10.8 *^,^#	30.2 ± 11.3	31.6 ± 11.5 #

Results shown as arithmetic mean ± standard deviation (SD). Abbreviations: NW, group participating in Nordic walking training; CG, control group; Mb, myoglobin; LDH, lactate dehydrogenase; Ca, total calcium; P, phosphorus; 25(OH)D_3_, calcidiol. * *p* > 0.05––statistically significant differences in a group, # *p* > 0.05––statistically significant differences between the NW and CG groups.

## Data Availability

Data are available on request from the corresponding author.
